# Retention of a cohort of men who have sex with men and transgender women at risk for and living with HIV in Abuja and Lagos, Nigeria: a longitudinal analysis

**DOI:** 10.1002/jia2.25592

**Published:** 2020-10-01

**Authors:** Blessing O Kayode, Andrew Mitchell, Nicaise Ndembi, Afoke Kokogho, Habib O Ramadhani, Sylvia Adebajo, Merlin L Robb, Stefan D Baral, Julie A Ake, Manhattan E Charurat, Trevor A Crowell, Rebecca G Nowak, the TRUST/RV368 Study Group, Manhattan Charurat, Julie Ake, Aka Abayomi, Sylvia Adebajo, Stefan Baral, Trevor Crowell, Charlotte Gaydos, Afoke Kokogho, Jennifer Malia, Olumide Makanjuola, Nelson Michael, Nicaise Ndembi, Rebecca Nowak, Oluwasolape Olawore, Zahra Parker, Sheila Peel, Habib Ramadhani, Merlin Robb, Cristina Rodriguez‐Hart, Eric Sanders‐Buell, Elizabeth Shoyemi, Sodsai Tovanabutra, Sandhya Vasan

**Affiliations:** ^1^ Institute of Human Virology Nigeria Abuja Nigeria; ^2^ Institute of Human Virology University of Maryland School of Medicine Baltimore MD USA; ^3^ HJF Medical Research International Abuja Nigeria; ^4^ U.S. Military HIV Research Program Walter Reed Army Institute of Research Silver Spring MD USA; ^5^ Maryland Global Initiatives Corporation‐ A University of Maryland Baltimore Affiliate Abuja Nigeria; ^6^ Henry M. Jackson Foundation for the Advancement of Military Medicine Bethesda MD USA; ^7^ Department of Epidemiology Johns Hopkins School of Public Health Baltimore MD USA

**Keywords:** sub‐Saharan Africa, retention in care, treatment adherence and compliance, sexual and gender minorities, HIV

## Abstract

**Introduction:**

Men who have sex with men (MSM), and transgender women (TGW), face specific obstacles to retention in care, particularly in settings with stigmatization such as sub‐Saharan Africa. We evaluated the impacts of HIV status and other factors on loss‐to‐follow‐up (LTFU) and visit adherence among MSM and TGW in Abuja and Lagos, Nigeria.

**Methods:**

TRUST/RV368 is an open cohort that provides comprehensive and integrated prevention and treatment services for HIV and sexually transmitted infections (STIs) at community venues supportive of sexual and gender minorities. Recruitment began in March 2013 and participants were followed every three months for up to 18 months. LTFU was defined as not presenting for an expected visit in the past 180 days. Visit adherence was calculated as a rate of completed visits adjusted by the number of three‐month intervals elapsed since enrolment. HIV and other factors predictive of LTFU and visit adherence were evaluated using Cox proportional hazards and Poisson regression models, respectively.

**Results:**

A total of 1447 participants who completed enrolment evaluations over two visits as of November 2018 were included in these analyses. Their median age was 24 years (interquartile range [IQR]: 21 to 28) and 53% (n = 766) were living with HIV. LTFU occurred in 56% (n = 808) and visit adherence was 0.62 (95% confidence interval: 0.61 to 0.64) visits per three‐month interval. Participants at risk and living with HIV had median follow‐up times of 12 months (IQR: 6 to 22), and 21 months (IQR: 12 to 30), respectively (*p* < 0.01). After controlling for other factors, LTFU was less common among participants living with HIV or other STIs and more common among those who did not own a cell phone, sold sex and had never undergone HIV testing prior to enrolment. These factors had parallel associations with visit adherence.

**Conclusions:**

Retention was suboptimal in Nigerian clinics designed to serve MSM and TGW. Particularly high LTFU and low visit adherence among participants at risk for HIV could complicate deployment of HIV prevention interventions. Marketing the benefits of testing, improving access to cell phones and nurturing more trust with clients may improve retention among marginalized communities in Nigeria.

## 1 INTRODUCTION

Retention of people at risk for and living with HIV (PLWH) in evidence‐based programmes has its challenges in countries across sub‐Saharan Africa where loss to follow‐up (LTFU) estimates range from 3% to 45% and are as high as 75% after linkage to treatment in Nigeria [1, 2]. In Nigeria and several other African countries, the criminalization of same‐sex sexual practices further hinders linkage and retention for sexual and gender minorities [3]. A recent meta‐analysis of African men who have sex with men (MSM) living with HIV showed that only 37% to 53% were taking antiretroviral therapy (ART) and 34% achieved viral suppression [4]. Sub‐optimal engagement and retention in care undermine the continent‐wide successes of expanded ART access for key populations.

The broad range of reported engagement and retention among MSM in sub‐Saharan Africa can be explained by both study‐level differences in assessing LTFU [1] as well as individual factors that predict LTFU such as younger age, transportation difficulties, lack of social support and overall perception of feeling healthy [5]. Men are also more difficult to retain than women due to a historical focus on HIV testing and treatment as a maternal and child health issue [6]. For sexual and gender minorities, sexual behaviour stigma further impedes access and retention in healthcare facilities [7]. Sexual behaviour stigma as a multifaceted construct includes enacted stigma (behavioural expressions including physical violence); internalized stigma (feelings of stigma) and anticipated stigma (expectations of stigma) [7, 8].

Community‐based models integrate members of distinct sub‐populations to mobilize care outside of traditional models or facilities of healthcare [9]. Such models circumvent many of the contextual challenges stigmatized individuals face. Merging of HIV prevention with MSM peer educators in Malawi enabled retention of 81% of 106 at‐risk MSM through three quarterly follow‐up visits, although many had previously demonstrated high adherence [10]. In Senegal, a stigma mitigation study sensitizing peer educators and healthcare workers at government health facilities reported a lower six‐month retention of 14% (102/724) [11]. In South Africa, two public health facilities designated for men‐only incorporated sensitized training on the sexual health needs of MSM and reported a two‐year retention of 82%, although less than 20% were MSM and retention estimates were restricted to those on ART [12]. Community‐based clinics for MSM, TGW and other sexual and gender minorities may mitigate stigma and social barriers, but it remains unclear whether they can retain a large cohort in HIV prevention and clinical care.

The TRUST/RV368 study, in conjunction with non‐governmental organizations, tailored their clinical care to thousands of Nigerian MSM and TGW. These facilities synergized health and human rights by housing an advocacy group and a health clinic with staff inclusive of sexual and gender minorities [13]. Together, facilities focused on the social, legal and sexual health needs of study participants and sensitization training was provided to promote integration of the two entities. Services included education about safer sex practices, distribution of condoms and condom‐compatible lubricants and diagnosis and treatment of HIV and other sexually transmitted infections (STIs) [14, 15, 16, 17, 18, 19]. Despite these services, we have previously reported a high HIV incidence of 15 infections per 100 person‐years in the cohort [20]. Incidence was highest among participants under 19 years of age, a group that has poor general engagement in the HIV prevention and care cascade [21]. We hypothesized that retention would be lower for those who were not living with HIV and characteristics more common among young MSM and TGW would be independently associated with a decrease in retention. Our objective was to identify factors associated with LTFU and visit adherence – two complementary measures of retention – among sexual and gender minorities in a community‐based HIV prevention and treatment study.

## 2 METHODS

### 2.1 Study design and population

TRUST/RV368 recruited biological males who reported sex with men in Abuja and Lagos, Nigeria, two urban centres approximately 530 km apart, into an open prospective HIV treatment‐as‐prevention study as previously described [22, 23]. In brief, respondent‐driven sampling was used by initiating well‐networked gender and sexual minorities, termed “seeds,” to provide referral coupons to three eligible peers, who similarly received three referral coupons upon enrolment. Participants were compensated with 1000 Naira (approximately $3 USD) for each peer referral.

Enrolment criteria included assigned male sex at birth, anal sex with a male partner in the past year, a valid referral coupon, and written informed consent in English or Hausa. Age inclusion criteria differed between sites based on Institutional Review Board (IRB) recommendations (≥16 years in Abuja or ≥18 years in Lagos). Participants were expected to present for a total of eight visits, beginning with enrolment evaluations spread over two visits approximately two weeks apart (visits 0 and 1). Subsequent visits (2 to 7) were scheduled at three‐month intervals for a total of 18 months. Participants received a monetary incentive for visit completion, starting at 1000 Naira upon enrolment and increasing by 200 Naira for each subsequent visit. Participants completed a structured questionnaire that captured demographic, behavioural and clinical characteristics, and provided urine samples, anal swabs and blood samples for HIV/STI diagnostics. Only those who completed the enrolment evaluations at least 180 days before data censoring, underwent an HIV test with a valid result, and did not relocate, die, or voluntarily withdraw from the study were included in these analyses.

### 2.2 Ethical considerations

IRBs at the Nigerian Federal Capital Territory Health Research Ethics Committee, the Nigerian Ministry of Defense in Nigeria, the University of Maryland Baltimore and the Walter Reed Army Institute of Research approved the research protocol. All participants provided informed consent.

### 2.3 Retention strategies

Prior to study implementation, staff underwent sensitization training that included a week‐long session with educators from the Fenway Institute, an internationally recognized interdisciplinary centre focused on the delivery of destigmatized medical care to MSM, TGW and PLWH. Ethnographic assessments, focus groups and meetings with grassroots organizations were conducted to educate staff on community needs. Phone numbers, emails and residential addresses were used to contact participants two weeks prior to each appointment. If an appointment was missed, staff attempted to reschedule with repeated phone calls or through social networks. During study visits, participants were escorted to each point of care to maximize comfort. To promote retention and minimize stigmatization, a Community Advisory Board comprised of sexual and gender minorities as well as other stakeholders, totalling 20 to 25 members, met as frequently as bi‐monthly to discuss the study procedures and environment at each clinic. Regular community social events, such as beauty pageants, candlelight processions, film screenings and panel discussions were organized to promote the clinics as safe spaces.

### 2.4 Laboratory procedures

At enrolment and subsequent visits, participants who were at risk for HIV underwent HIV testing using fingerstick collection of whole blood and Determine (Alere, Waltham, MA, USA) and Uni‐gold (Trinity biotech, Co‐Wicklow, Ireland) test kits as outlined by the parallel testing algorithm [24]. A third rapid test, HIV‐1/2 Stat‐Pak (Chembio Diagnostics, Medford, NY, USA) was used for discordant results. For all participants, voided urine and anal swabs were tested for *Neisseria gonorrhoeae* (NG) and *Chlamydia trachomatis* (CT) using the Aptima Combo 2 CT/NG Assay (Hologic, San Diego, CA, USA). Participants found to be living with HIV underwent ART preparation and initiation per treatment‐as‐prevention guidelines [22]. Bacterial STIs were treated with antibiotic therapy provided at the clinics.

### 2.5 Outcomes

#### 2.5.1 Loss to follow‐up

Under a prospective definition of LTFU, participants who do not present for a minimum number of days or study visits are categorized as LTFU, regardless of whether they ultimately re‐engage. This method often results in misclassification of individuals who return to the facility after an extended absence [1]. To avoid misclassification, we categorized participants based on number of days since their most recent visit, regardless of intermittent gaps in care. These gaps may have exceeded 180 days, but participants were not considered LTFU if they eventually returned to clinic. Participants who completed all scheduled visits were censored at their last visit and those in ongoing follow‐up were censored on 2 November 2018.

#### 2.5.2 Visit adherence

To account for varying periods of study observation, visit adherence was calculated by dividing the number of completed visits by the number of expected visits. This was expressed as a rate of visits completed per three‐month interval, which was the expected interval between scheduled visits.

### 2.6 Independent variables

HIV status was the main exposure of interest. Demographic and behavioural characteristics were also explored as independent predictors of LTFU and visit adherence. All predictors were assessed at enrolment in order to replicate the risk stratification and profiling that occur at initial entry into care. This did not allow for covariates that changed during follow‐up, such as in the case of HIV seroconversion. Covariates included age, study site, education level, employment status, sexual orientation, gender identity, cell phone ownership, number of male sexual partners in the past year, insertive and/or receptive sexual practices (IAI, RAI), condom use with a male partner at last anal sex, buying and/or selling of sex, prior HIV testing and worry about HIV infection. For gender, participants were asked “What do you consider your gender to be?”. Options included man, woman, other or both man and woman, and participants were categorized as cisgender men, TGW or other/unknown gender. Characteristics related to stigma included disclosure of MSM status to healthcare workers and sexual behaviour stigma (self‐reported experience of verbal harassment as a result of being MSM and fear of accessing healthcare services because of worry someone may learn MSM status). Social support characteristics were captured on a 4‐point Likert scale (1 = strongly disagree to 4 = strongly agree) and dichotomized as any agreement vs. any disagreement to the following statements: “The group of friends with whom you socialize is a mix of straight people and MSM,” and “You can trust the majority of MSM you know.” HIV, CT and NG status at enrolment were based on study‐provided laboratory testing.

### 2.7 Statistical analysis

LTFU rates were calculated for all participants and within groups of each characteristic. For our primary analyses, the youngest age group considered as an independent variable in models was 16 to 19 years; since only the Abuja site enrolled participants aged 16 to 17 years, we compared characteristics of the 16 to 17 year and 18 to 19 year groups from Abuja using Chi‐square tests. For LTFU, Cox proportional hazards regression models were used to identify predictive characteristics in bivariate analyses (*p* < 0.05), and a multivariable model was built using forward stepwise selection with priority given to factors previously associated with LTFU. Remaining factors were entered according to the magnitude of their crude association with LTFU, and all that remained significant were retained in the model.

Poisson regression models of visit adherence were offset by the log of expected number of visits according to time elapsed since date of enrolment, and resulting coefficients were exponentiated into rate ratios. Similar to LTFU analyses, candidate variables identified in bivariate analyses were entered into a multivariable model via forward stepwise selection. Crude and adjusted rate ratios with 95% confidence intervals (CIs) were calculated for each characteristic.

Analyses were repeated with stratification by gender to evaluate differences in correlates of retention among MSM and TGW. Data were analysed using Statistical Analysis Software (SAS) version 9.4 (SAS Institute, Cary, NC).

## 3 RESULTS

A total of 2,386 participants enrolled in the TRUST/RV368 cohort from March 2013 to November 2018. Twenty‐one percent (n = 491) were excluded because of a missing HIV test result, 14% (n = 323) did not complete both enrolment visits, 4% (n = 107) enrolled less than 180 days before data censoring and <1% relocated (n = 9), withdrew (n = 5) or died (n = 4).

A total of 1447 participants were included in these analyses with median age 24 years (interquartile range [IQR]:21 to 28). PLWH had median age 26 years (IQR: 23 to 29), whereas participants at risk for HIV had median age 23 years (IQR: 20 to 27; *p* < 0.01). Eighty percent (n = 1153) identified as cisgender men, and 43% (n = 618) sold sex within the past year (Table 1). Enrolment prevalence of HIV, CT and NG was 53% (n = 766), 16% (n = 235) and 21% (n = 304) respectively. A significantly lower proportion of 16 to 17 year‐olds owned a cell phone, used a condom at last anal sex, ever tested for HIV, and were afraid to access health services as compared to 18 to 19 year olds in Abuja (all *p* < 0.05) (Table S1).

**Table 1:** Distribution of demographic, behavioural and clinical characteristics of participants enrolled in TRUST/RV368 overall and by HIV status

Characteristic	Total N = 1447 n (%)	At risk for HIV N = 681 n (%)	PLWH N = 766 n (%)	*p* value
Age (years)				<0.01
16 to 19	197 (13.6)	135 (19.8)	62 (8.1)	
20 to 24	603 (41.7)	294 (43.2)	309 (40.3)	
25+	647 (44.7)	252 (37.0)	395 (51.6)	
Study site				<0.01
Abuja	986 (68.1)	522 (76.7)	464 (60.6)	
Lagos	461 (31.9)	159 (23.3)	302 (39.4)	
Education				0.11
≤ High school	914 (63.2)	445 (65.3)	469 (61.3)	
> High school	532 (36.8)	236 (34.7)	296 (38.7)	
Employment status				0.62
Unemployed	352 (24.8)	168 (25.4)	184 (24.2)	
Employed/student	1069 (75.2)	494 (74.6)	575 (75.8)	
Sexual orientation				0.02
Homosexual	453 (31.4)	192 (28.3)	261 (34.2)	
Bisexual	988 (68.6)	486 (71.7)	502 (65.8)	
Gender identity				<0.01
Cisgender man	1153 (79.9)	571 (84.1)	582 (76.2)	
Transgender woman	158 (10.9)	60 (8.8)	98 (12.8)	
Other/unknown	132 (9.1)	48 (7.1)	84 (11.0)	
Owns a cell phone				<0.01
No	69 (4.8)	49 (7.2)	20 (2.6)	
Yes	1371 (95.2)	629 (92.8)	742 (97.4)	
Number of male sexual partners in past year				<0.01
0 to 4	691 (48.3)	354 (52.8)	337 (44.2)	
5 to 9	379 (26.5)	175 (26.1)	204 (26.8)	
10+	362 (25.3)	141 (21.0)	221 (29.0)	
Receptive and/or insertive anal sexual practices in the past year				<0.01
RAI	309 (21.7)	123 (18.5)	186 (24.5)	
IAI	351 (24.6)	247 (37.1)	104 (13.7)	
IAI and RAI	766 (53.7)	296 (44.4)	470 (61.8)	
Condom used at last anal sex with male partner				0.11
No	498 (34.6)	249 (36.7)	249 (32.7)	
Yes	943 (65.4)	430 (63.3)	513 (67.3)	
Sold sex in past year				0.09
No	818 (57.0)	368 (54.6)	450 (59.1)	
Yes	618 (43.0)	306 (45.4)	312 (40.9)	
Bought sex in past year				0.27
No	1032 (71.9)	493 (73.3)	539 (70.6)	
Yes	404 (28.1)	180 (26.7)	224 (29.4)	
Ever test for HIV				<0.01
Yes	1165 (80.7)	502 (73.9)	663 (86.7)	
At least somewhat worried about HIV in the past year				0.04
No	917 (63.6)	449 (66.4)	468 (61.1)	
Yes	525 (36.4)	227 (33.6)	298 (38.9)	
Ever been verbally harassed for being MSM				<0.01
No	1000 (69.2)	515 (75.7)	485 (63.3)	
Yes	446 (30.8)	165 (24.3)	281 (36.7)	
Ever disclosed MSM status to healthcare worker				<0.01
No	923 (64.0)	500 (73.9)	423 (55.3)	
Yes	519 (36.0)	177 (26.1)	342 (44.7)	
Ever been afraid to access health services				<0.01
No	936 (64.7)	474 (69.6)	462 (60.4)	
Yes	510 (35.3)	207 (30.4)	303 (39.6)	
Friends with whom socialize are MSM and heterosexual				0.95
No	90 (6.2)	42 (6.2)	48 (6.3)	
Yes	1351 (93.8)	635 (93.8)	716 (93.7)	
Trusts the majority of other MSM they know				<0.01
No	686 (47.6)	274 (40.5)	412 (53.9)	
Yes	755 (52.4)	402 (59.5)	353 (46.1)	
*Chlamydia trachomatis*				0.34
Negative	1196 (83.6)	555 (82.6)	641 (84.5)	
Positive	235 (16.4)	117 (17.4)	118 (15.5)	
*Neisseria gonorrhoeae*				<0.01
Negative	1127 (78.8)	552 (82.1)	575 (75.8)	
Positive	304 (21.2)	120 (17.9)	184 (24.2)	

IAI, insertive anal intercourse; MSM, men who have sex with men; PLWH, people living with HIV; RAI, receptive anal intercourse.

Median follow‐up time in the cohort was 17.3 months (IQR: 8 to 26 months) and participants completed a median 71% of their expected visits (IQR: 25 to 100%). Participants at risk for HIV had a median follow‐up time of 12.4 months (IQR: 6 to 22 months), compared to 20.5 months (IQR: 12 to 30 months) for PLWH (*p* < 0.01). Participants at risk for HIV had a median proportion of visit adherence of 42.9% (IQR: 14% to 86%), compared to 85.7% (IQR: 40% to 100%) for PLWH (*p* < 0.01).

Over the course of the study 56% (n = 808) of participants were LTFU. This included 66% (n = 449) of all participants at risk for HIV and 47% (n = 359) of all PLWH. Being at risk for HIV was independently associated with increased risk of LTFU (HR: 1.72, 95% CI: 1.49 to 2.00; Table 2) and lower visit adherence (RR: 0.80, 95% CI: 0.75 to 0.85; Table 3). Younger age was not predictive of LTFU or visit adherence in the multivariable models, though participants in the 20‐ to 24‐year‐old group had lower visit adherence (RR: 0.92, 95% CI: 0.87, 0.98) and a non‐significant increase in LTFU (HR: 1.14, 95% CI: 0.97 to 1.34) as compared to participants 25 years or older. Exploratory predictors of retention consistent across models for both LTFU and visit adherence included not owning a cell phone, selling sex within the past year, no prior testing for HIV, trusting the majority of MSM acquaintances, and not presenting with NG at enrolment (Tables 2 and 3). Belonging to a social group comprised of MSM and heterosexuals was significantly associated with increased visit adherence but not LTFU. After stratification by gender, participants at risk for HIV had lower retention among cisgender MSM and participants with other/unknown gender, but not TGW. All exploratory predictors identified earlier were also significant in analyses restricted to cisgender MSM (Tables S1 and S2). For TGW, owning a cell phone or prior testing for HIV were the only significant predictors of retention (Tables S4 and S5). For participants with other/unknown gender, living with HIV was the only independent predictor of retention (Tables S6 and S7).

**Table 2:** Loss‐to‐follow‐up among participants enrolled in TRUST/RV368 by demographic, behavioural and clinical characteristics

Characteristic	LTFU n	PY	LTFU rate	Crude HR (95% CI)	*p‐*value	Adjusted HR (95% CI)	*p‐*value
Overall	808	2471	32.70	–	–		
HIV status							
At risk	449	975	46.03	**1.95 (1.69** to **2.24)**	<.01	**1.72 (1.49** to **2.00)**	<.01
Living with HIV	359	1495	24.01	ref	–	–	–
Age (years)							
16 to 19	125	298	41.90	**1.51 (1.23** to **1.86)**	<0.01	1.09 (0.86 to 1.37)	0.48
20 to 24	353	993	35.55	**1.31 (1.12** to **1.52)**	<0.01	1.14 (0.97 to 1.34)	0.10
25+	330	1179	27.98	ref	–	–	–
Study site							
Abuja	538	1639	32.82	ref	–		
Lagos	270	831	32.47	0.99 (0.85 to 1.15)	0.87		
Education							
≤High school	536	1491	35.94	**1.32 (1.14** to **1.52)**	<0.01		
>High school	271	977	27.75	ref	–		
Employment status							
Unemployed	180	592	30.40	ref	–		
Employed/student	602	1863	32.31	1.04 (0.88 to 1.23)	0.62		
Sexual orientation							
Homosexual	246	795	30.94	ref	–		
Bisexual	557	1668	33.40	1.08 (0.93 to 1.25)	0.32		
Gender identity							
Cisgender man	663	1966	33.73	ref	–		
Transgender woman	81	275	29.45	0.88 (0.70 to 1.11)	0.29		
Other/unknown	61	222	27.44	0.84 (0.64 to 1.09)	0.18		
Owns a cell phone							
No	51	73	69.88	**2.30 (1.73** to **3.06)**	<0.01	**1.82 (1.35** to **2.45)**	<0.01
Yes	752	2384	31.55	ref	–	–	–
Number of male sexual partners in past year							
0 to 4	367	1147	32.00	ref	–		
5 to 9	214	640	33.46	1.07 (0.90 to 1.26)	0.45		
10+	212	672	31.56	0.99 (0.84 to 1.17)	0.91		
Receptive and/or insertive anal sexual practices in the past year							
RAI only	155	546	28.38	ref			
IAI only	216	591	36.54	**1.28 (1.04** to **1.57)**	0.02		
IAI and RAI	418	1312	31.87	1.13 (0.94 to 1.36)	0.18		
Condom used at last anal sex with male partner							
No	285	855	33.32	1.04 (0.90 to 1.20)	0.59		
Yes	517	1605	32.22	ref	–		
Sold sex in the past year							
No	415	1467	28.29	ref	–	–	–
Yes	383	995	38.50	**1.38 (1.20** to **1.58)**	<0.01	**1.34 (1.15** to **1.55)**	<0.01
Bought sex in the past year							
No	574	1773	32.37	ref			
Yes	224	691	32.42	1.01 (0.86 to 1.18)	0.93		
Ever tested for HIV							
No	206	419	49.21	**1.72 (1.46** to **2.01)**	<0.01	**1.46 (1.23** to **1.73)**	<0.01
Yes	600	2048	29.29	ref	–	–	–
At least somewhat worried about HIV in past year							
No	525	1557	33.72	1.11 (0.96 to 1.23)	0.16		
Yes	278	910	30.55	ref	–		
Ever been verbally harassed for being MSM							
No	540	1652	32.69	ref	–		
Yes	267	819	32.62	0.98 (0.85 to 1.14)	0.83		
Ever disclosed MSM status to healthcare worker							
No	516	1503	34.34	**1.18 (1.02** to **1.37)**	0.02		
Yes	287	965	29.75	ref	–		
Ever been afraid to access health services							
No	529	1589	33.28	1.06 (0.92 to 1.23)	0.40		
Yes	279	880	31.69	ref	–		
Friends with whom socialize are MSM and heterosexual							
No	63	162	38.86	ref	–		
Yes	739	2304	32.08	0.83 (0.64 to 1.08)	0.17		
Trusts the majority of other MSM they know							
No	344	1257	27.38	ref	–	–	–
Yes	458	1210	37.86	**1.38 (1.20** to **1.58)**	<0.01	**1.30 (1.13** to **1.51)**	<0.01
*Chlamydia trachomatis*							
Negative	667	2048	32.56	1.05 (0.87 to 1.27)	0.59		
Positive	128	413	31.00	ref	–		
*Neisseria gonorrhoeae*							
Negative	647	1849	34.98	**1.45 (1.21** to **1.73)**	<0.01	**1.47 (1.22** to **1.77)**	<0.01
Positive	148	612	24.19	ref	–	–	–

Bolding indicates *p* < 0.05.

CI, confidence interval; HR, hazard ratio; IAI, insertive anal intercourse; LTFU, loss‐to‐follow‐up; MSM, men who have sex with men; PY, person‐years; RAI, receptive anal intercourse.

**Table 3:** Visit adherence among participants enrolled in TRUST/RV368 by demographic, behavioural and clinical characteristics

Characteristic	Visit adherence rate (95% CI)	Crude RR (95% CI)	*p* value	Adjusted RR (95% CI)	*p* value
Overall	0.62 (0.61 to 0.64)	–	–		
HIV status					
At risk	0.51 (0.49 to 0.54)	**0.72 (0.68** to **0.75)**	<0.01	**0.80 (0.75** to **0.85)**	<0.01
Living with HIV	0.72 (0.69 to 0.74)	ref	–	–	–
Age (years)					
16 to 19	0.55 (0.51 to 0.59)	**0.82 (0.75** to **0.88)**	<0.01	0.95 (0.87 to 1.04)	0.28
20 to 24	0.60 (0.57 to 0.62)	**0.89 (0.84** to **0.94)**	<0.01	**0.92 (0.87** to **0.98)**	<0.01
25+	0.67 (0.65 to 0.70)	ref	–	–	–
Study site					
Abuja	0.59 (0.57 to 0.60)	ref	–		
Lagos	0.70 (0.67 to 0.73)	**1.19 (1.13** to **1.25)**	<0.01		
Education					
≤High school	0.59 (0.57 to 0.61)	**0.87 (0.83** to **0.92)**	<0.01		
>High school	0.68 (0.65 to 0.70)	ref	–		
Employment status					
Unemployed	0.65 (0.62 to 0.78)	ref	–		
Employed/student	0.62 (0.60 to 0.64)	0.96 (0.91 to 1.02)	0.18		
Sexual orientation					
Homosexual	0.65 (0.62 to 0.68)	ref	–		
Bisexual	0.61 (0.59 to 0.63)	**0.94 (0.89** to **0.99)**	0.02		
Gender					
Cisgender man	0.61 (0.59 to 0.63)	ref	–		
Transgender woman	0.66 (0.62 to 0.71)	**1.09 (1.00** to **1.17)**	0.04		
Other/unknown	0.66 (0.61 to 0.72)	1.09 (0.99 to 1.19)	0.06		
Owns a cell phone					
No	0.42 (0.36 to 0.48)	**0.66 (0.57** to **0.77)**	<0.01	**0.81 (0.70** to **0.94)**	<0.01
Yes	0.63 (0.62 to 0.65)	ref	–	–	–
Number of male sexual partners in the past year					
0 to 4	0.63 (0.60 to 0.65)	ref	–		
5 to 9	0.62 (0.59 to 0.75)	0.99 (0.94 to 1.06)	0.87		
10+	0.64 (0.60 to 0.67)	1.01 (0.95 to 1.08)	0.66		
Receptive and/or insertive anal sexual practices in the past year					
RAI only	0.67 (0.64 to 0.71)	ref	–	–	–
IAI only	0.58 (0.55 to 0.61)	**0.86 (0.80** to **0.93)**	<0.01	**0.89 (0.82** to **0.97)**	<0.01
IAI and RAI	0.63 (0.61 to 0.66)	0.95 (0.89 to 1.01)	0.08	**0.92 (0.87** to **0.98)**	0.02
Condom used at last anal sex with male partner					
No	0.61 (0.58 to 0.63)	0.96 (0.91 to 1.01)	0.10		
Yes	0.63 (0.61 to 0.65)	ref	–		
Sold sex in the past year					
No	0.66 (0.64 to 0.69)	ref	–	–	–
Yes	0.57 (0.55 to 0.60)	**0.86 (0.82** to **0.91)**	<0.01	**0.90 (0.85** to **0.95)**	<0.01
Bought sex in the past year					
No	0.63 (0.61 to 0.65)	ref	–		
Yes	0.61 (0.58 to 0.64)	0.97 (0.91 to 1.03)	0.27		
Ever tested for HIV					
No	0.45 (0.42 to 0.48)	**0.67 (0.63** to **0.73)**	<0.01	**0.74 (0.69** to **0.80)**	<0.01
Yes	0.67 (0.65 to 0.68)	ref	–	–	–
At least somewhat worried about HIV in the past year					
No	0.61 (0.59 to 0.63)	0.96 (0.91 to 1.01)	0.11		
Yes	0.64 (0.62 to 0.67)	ref	–		
Ever been verbally harassed for being MSM					
No	0.62 (0.60 to 0.64)	ref	–		
Yes	0.64 (0.61 to 0.67)	1.03 (0.98 to 1.09)	0.25		
Ever disclosed MSM status to healthcare worker					
No	0.60 (0.58 to 0.62)	**0.90 (0.85** to **0.97)**	<0.01		
Yes	0.67 (0.64 to 0.69)	ref	–		
Ever been afraid to access health services					
No	0.60 (0.58 to 0.62)	**0.92 (0.87** to **0.97)**	<0.01		
Yes	0.66 (0.63 to 0.68)	ref	–		
Friends with whom socialize are MSM and heterosexual					
No	0.53 (0.47 to 0.59)	ref	–	–	–
Yes	0.63 (0.61 to 0.65)	**1.20 (1.07** to **1.34)**	<0.01	**1.15 (1.02** to **1.28)**	0.02
Trusts the majority of other MSM they know					
No	0.68 (0.65 to 0.70)	ref	–	–	–
Yes	0.57 (0.55 to 0.60)	**0.85 (0.81** to **0.89)**	<0.01	**0.90 (0.85** to **0.94)**	<0.01
*Chlamydia trachomatis*					
Negative	0.62 (0.61 to 0.64)	0.97 (0.90 to 1.04)	0.34		
Positive	0.64 (0.61 to 0.68)	ref	–		
*Neisseria gonorrhoeae*					
Negative	0.61 (0.59 to 0.62)	**0.87 (0.82** to **0.92)**	<0.01	**0.89 (0.83** to **0.94)**	<0.01
Positive	0.70 (0.66 to 0.74)	ref	–	–	–

Bolding indicates *p* < 0.05.

CI, confidence interval; IAI, insertive anal intercourse; MSM, men who have sex with men; RAI, receptive anal intercourse; RR, risk ratio.

## 4 DISCUSSION

HIV status was a significant predictor of both LTFU and visit adherence in our study. While PLWH had better retention outcomes, overall retention for all groups evaluated in this study was suboptimal. Prior retention estimates for MSM in Malawi and Senegal were 81% and 14% over six or nine months, respectively [10, 11], a wide range that brackets our estimate of 44% despite shorter follow‐up. Our annualized LTFU rate of 32.7 per 100 person‐years was lower than the 59.5 per 100 person‐years observed in a retrospective cohort study of reproductive aged adults attending PEPFAR‐supported pre‐ART programmes in Nigeria [2]. Variable measures and a paucity of data on retention in sub‐Saharan Africa complicate contextualization of our findings, but our study shows clear room for improvement in retaining MSM and TGW in HIV prevention and care.

Younger age did not predict retention, but there were some downstream factors indicative of youth that were associated with poor retention. For example among our youngest participants, 16 to 17 year‐olds were significantly less likely to own a cell phone as compared to 18 to 19 year‐olds in Abuja. Owning a cell phone was associated with better retention in our study, which could be explained by the fact that phones were a primary mode of contact for reminders of upcoming and missed appointments. For participants without cell phones or working phone numbers, clinic staff relied on social networks to maintain contact. To reach young participants without phones, a peer‐mentoring programme could be employed to facilitate social network‐based communication [25]. Social media may be another avenue, as even those without cell phones may be able to access the internet and social media accounts through shared devices.

Individuals who had not been tested for HIV prior to study enrolment were at higher risk of LTFU and less likely to adhere to the visit schedule. For many, prior avoidance of healthcare engagement for HIV testing may have been driven by anticipated stigma [26, 27, 28, 29, 30]. Other qualitative studies have shown that sexual and gender minorities avoid testing because a diagnosis of HIV or rectal STIs could lead to unintended disclosure of anal sex practices [4, 31, 32]. We have previously reported substantial anticipated and enacted stigma surrounding disclosure of same sex sexual practices by participants in our cohort [7, 33, 34, 35]. Despite the goal of non‐stigmatizing care delivery in our clinics, it is possible that participants who had not previously been tested for HIV had experiences that affirmed their anticipated stigma and reduced likelihood of retention. Alternatively, non‐stigma‐related characteristics such as being young and having a lower risk perception, which may have been confirmed with negative HIV/STI test results, could have impeded retention [5]. Further qualitative evaluation of factors that influence retention could be valuable to tailor retention strategies.

Prior studies have shown that men can be particularly difficult to retain in HIV care [36, 37, 38, 39], and while most prior studies focused on heterosexual and cisgender adults, the same gender norms may play a role in retention for MSM and TGW. A qualitative study in eastern Africa suggested that men perceived health clinics as women’s spaces and engaging in care was more of a concern and activity of women [40]. In our study, living with HIV was independently associated with improved retention among cisgender men and participants with other/unknown gender, but HIV status did not predict retention among TGW. Additional research examining perceptions of health clinics among TGW in sub‐Saharan Africa could lend insight to this non‐effect.

Healthcare engagement can also be facilitated by concern about specific signs, symptoms, or diagnoses rather than prevention of ailments that are yet to occur. Our findings suggested higher retention of participants with a diagnosis of HIV or STIs at enrolment, likely driven by their need for treatment. When a person feels healthy or tests negative for HIV, active engagement in care tends to dissipate [41]. In prior studies, higher attrition has been observed among PLWH with indicators of asymptomatic disease, such as high CD4 counts [42] or no AIDS‐defining diagnosis [43]. Education about preventive medical needs may be needed to shift perceptions on accessing healthcare to improve retention for individuals at risk for HIV and other STIs.

Transactional sex involves a culmination of factors that contribute to a lack of engagement such as stigmas from commercial sex work and same‐sex sexual practices as well as power imbalances and socio‐economic vulnerability [44, 45, 46]. These may co‐occur with mental health challenges, low self‐esteem and withdrawal [35, 47]. Sexual and gender minorities with many of these vulnerabilities [48, 49] may be less inclined to attend visits regularly. Our participants who engaged in transactional sex were also younger [45], reinforcing further disengagement because adolescents have a lower perceived risk of HIV infection [45, 50]. Incorporating peer‐led social groups at the clinics to promote trust and agency may increase retention, similar to what has been done for female sex workers in Zimbabwe [51].

This study has some limitations. First, understanding the interplay between HIV status, sexual behaviours, stigma and retention required test results and completion of both enrolment questionnaires, which were unavailable for 491 and 323 participants, respectively. This may have resulted in an underestimate of overall LTFU, including a substantial minority of participants who were lost between visits 0 and 1 but unevaluable because of incomplete data. Second, this study could not assess whether lost participants re‐engaged in care elsewhere [52]. If re‐engagement occurred, then we overestimated the true loss and underestimated overall retention. Third, a proportion of men seroconverted and the diagnosis of HIV may have altered their engagement with the clinic over time, likely leading to overestimated retention for participants at risk for HIV. The unavailability of pre‐exposure prophylaxis medication in Nigeria at the time of this study should also be noted in context of our retention estimates. Enrolment characteristics were used in analyses rather than time‐varying ones to allow for comparability with future prospective studies, which must determine eligibility and anticipate retention based on initial evaluations. However, retention may have been influenced by characteristics that changed over time. Finally, this study originated in two cities in Nigeria and may not be generalizable to other areas, or on a national scale.

## 5 CONCLUSIONS

Overall, retention in this study was suboptimal but within the range of other regional and continental estimates. Participants living with HIV demonstrated better retention as compared to those at risk for HIV, although HIV status did not impact retention specifically for TGW in this study. Marketing the benefits of testing, improving access to cell phones and nurturing trust with clients may further improve retention among marginalized Nigerian MSM and TGW. Clinic‐level interventions to improve retention must be accompanied by rights‐affirming structural interventions to maximize benefits to key populations in Nigeria.

## COMPETING INTEREST

The authors have no conflicts of interest.

## AUTHORS’ CONTRIBUTIONS

BK, AM and RN conceived the analysis for the manuscript. Data collection and management was facilitated by BK, AK, NN and HR. AM conducted the data analysis with input from RN and TC. BK, AM and RN drafted the manuscript and AK, NN, HR, SA, MR, SB, JA, MC and TC provided critical review and editing. All authors have seen and approved the paper.

## Supporting information


**Table S1.** Distribution of demographic, behavioral and clinical characteristics of young participants enrolled in TRUST/RV368* by age group
**Table S2.** Loss‐to‐follow‐up among participants enrolled in TRUST/RV368 by demographic, behavioral, and clinical characteristics: cisgender men
**Table S3.** Visit adherence among participants enrolled in TRUST/RV368 by demographic, behavioral, and clinical characteristics: cisgender men
**Table S4.** Loss‐to‐follow‐up among participants enrolled in TRUST/RV368 by demographic, behavioral, and clinical characteristics: transgender women
**Table S5.** Visit adherence among participants enrolled in TRUST/RV368 by demographic, behavioral, and clinical characteristics: transgender women
**Table S6.** Loss‐to‐follow‐up among participants enrolled in TRUST/RV368 by demographic, behavioral, and clinical characteristics: other or unknown gender
**Table S7.** Visit adherence among participants enrolled in TRUST/RV368 by demographic, behavioral, and clinical characteristics: other or unknown gender

## Data Availability

The data that support the findings of this study are available upon request from the TRUST/RV368 Study Team. As a precautionary measure due to the criminalization of same‐sex behaviour in Nigeria, the research data are kept confidential.
