# Supplementary material for: Retention of a cohort of men who have sex with men and transgender women at risk for and living with HIV in Abuja and Lagos, Nigeria: a longitudinal analysis
Source: J Int AIDS Soc. 2020 Oct 1;23(Suppl 6):e25592. doi: 10.1002/jia2.25592 (PMC7527765; doi:10.1002/jia2.25592)
Supplement: Supplementary file 1 — Table S1. Distribution of demographic, behavioral and clinical characteristics of young participants enrolled in TRUST/RV368* by age group Table S2. Loss‐to‐follow‐up among participants enrolled in TRUST/RV368 by demographic, behavioral, and clinical characteristics: cisgender men Table S3. Visit adherence among participants enrolled in TRUST/RV368 by demographic, behavioral, and clinical characteristics: cisgender men Table S4. Loss‐to‐follow‐up among participants enrolled in TRUST/RV368 by demographic, behavioral, and clinical characteristics: transgender women Table S5. Visit adherence among participants enrolled in TRUST/RV368 by demographic, behavioral, and clinical characteristics: transgender women Table S6. Loss‐to‐follow‐up among participants enrolled in TRUST/RV368 by demographic, behavioral, and clinical characteristics: other or unknown gender Table S7. Visit adherence among participants enrolled in TRUST/RV368 by demographic, behavioral, and clinical characteristics: other or unknown gender [file JIA2-23-e25592-s001.docx]

| *Supplemental Table 1. Distribution of demographic, behavioral and clinical characteristics of young participants enrolled in TRUST/RV368* by age group* | | | |
| --- | --- | --- | --- |
| **Characteristic** | **Age 16-17**  n=29  n (%) | **Age 18-19**  n=83  n (%) | ***p*** |
| HIV |  |  | 0.06 |
| At risk  Living with HIV | 27 (93.2)  2 (6.8) | 64 (77.2)  19 (22.8) |  |
| Education  ≤ high school  > high school | 28 (96.6)  1 (3.4) | 73 (88.0)  10 (12.0) | 0.18 |
| Employment status  Unemployed  Employed/student | 7 (25.0)  21 (75.0) | 29 (36.2)  51 (63.8) | 0.28 |
| Sexual orientation  Homosexual  Bisexual | 10 (34.4)  19 (65.6) | 29 (35.0)  51 (63.8) | 0.96 |
| Gender identity  Cisgender man  Transgender woman  Other/unknown | 23 (79.4)  3 (10.4)  3 (10.4) | 71 (85.6)  9 (10.8)  3 (3.6) | 0.38 |
| Owns a cell phone  No  Yes | 9 (31.0)  20 (69.0) | 6 (7.2)  77 (92.8) | <0.01 |
| Number of male sexual partners in past year | | | 0.23 |
| 0-4  5-9  10+ | 9 (32.2)  13 (46.4)  6 (21.4) | 39 (48.2)  24 (29.6)  18 (22.2) |  |
| Receptive and/or insertive anal sexual practices in the past year | | | 0.29 |
| RAI  IAI  IAI and RAI | 8 (28.6)  3 (10.8)  17 (60.8) | 30 (38.4)  14 (18.0)  34 (43.6) |  |
| Condom used at last anal sex with male partner | | | <0.01 |
| No  Yes | 16 (55.2)  13 (44.8) | 22 (26.6)  61 (73.4) |  |
| Sold sex in past year | | | 0.37 |
| No  Yes | 9 (31.0)  20 (69.0) | 32 (40.6)  47 (59.4) |  |
| Bought sex in past year | | | 0.75 |
| No  Yes | 24 (82.8)  5 (17.2) | 64 (80.0)  16 (20.0) |  |
| Ever tested for HIV | | | <0.01 |
| No  Yes | 18 (62.0)  11 (38.0) | 27 (32.6)  56 (67.4) |  |
| At least somewhat worried about HIV in the past year | | | 0.32 |
| No  Yes | 21 (72.4)  8 (27.6) | 51 (62.2)  31 (37.8) |  |
| Ever been verbally harassed for being MSM | | | 0.07 |
| No  Yes | 19 (65.6)  10 (34.4) | 68 (82.0)  15 (18.0) |  |
| Ever disclosed MSM status to healthcare worker | | | 0.97 |
| No  Yes | 25 (89.2)  3 (10.8) | 73 (89.0)  9 (11.0) |  |
| Ever been afraid to access health services | | | 0.04 |
| No  Yes | 25 (86.2)  4 (13.8) | 55 (66.2)  28 (33.8) |  |
| Friends with whom socialize are MSM and heterosexual | | | 0.25 |
| No  Yes | 2 (7.2)  26 (92.8) | 2 (2.4)  80 (97.6) |  |
| Trusts the majority of other MSM they know | |  | 0.25 |
| No  Yes | 7 (25.0)  21 (75.0) | 30 (37.0)  51 (63.0) |  |
| *Chlamydia trachomatis* | | | 0.06 |
| Negative  Positive | 27 (82.6)  2 (17.4) | 64 (77.2)  19 (22.8) |  |
| *Neisseria gonorrhoeae* | | | 0.11 |
| Negative  Positive | 25 (86.2)  4 (13.8) | 59 (71.0)  24 (29.0) |  |
| Abbreviations: p, p-value; RAI, receptive anal intercourse; IAI, insertive anal intercourse; MSM, men who have sex with men.  *Includes only participants enrolled in Abuja site | | | |

| *Supplemental Table 2. Loss-to-follow-up among participants enrolled in TRUST/RV368 by demographic, behavioral, and clinical characteristics: cisgender men* | | | | | | | | |
| --- | --- | --- | --- | --- | --- | --- | --- | --- |
|  |  |  |  |  |  |  |  |  |
| **Characteristic** | | **LTFU n** | **PY** | **LTFU rate** | **Crude**  **HR (95% CI)** | ***p*** | **Adjusted**  **HR (95% CI)** | ***p*** |
| Overall | | 663 | 1966 | 33.73 | -- | -- |  |  |
| HIV | | | | | | | | |
|  | At risk | 388 | 830 | 46.75 | **1.96 (1.68, 2.29)** | <.01 | **1.72 (1.49, 2.00)** | <.01 |
|  | Living with HIV | 275 | 1136 | 24.21 | ref | -- | -- | -- |
| Age (years) | |  |  |  |  |  |  |  |
|  | 16-19 | 103 | 229 | 45.04 | **1.57 (1.25, 1.97)** | <.01 | 1.15 (0.89, 1.48) | 0.30 |
|  | 20-24 | 279 | 772 | 36.16 | **1.28 (1.08, 1.51)** | <.01 | 1.13 (0.95, 1.35) | 0.17 |
|  | 25+ | 281 | 966 | 29.10 | ref | -- | -- | -- |
| Study site | | | | | | | | |
|  | Abuja | 446 | 1323 | 33.70 | ref | -- |  |  |
|  | Lagos | 217 | 643 | 37.77 | 1.01 (0.85, 1.10) | 0.95 |  |  |
| Education | | | | | | | | |
|  | ≤ high school | 436 | 1149 | 37.96 | **1.39 (1.18, 1.63)** | <.01 |  |  |
|  | > high school | 227 | 817 | 27.77 | ref | -- |  |  |
| Employment status | | | | | | | | |
|  | Unemployed | 142 | 457 | 31.04 | ref | -- |  |  |
|  | Employed/ student | 500 | 1496 | 33.43 | 1.06 (0.88, 1.27) | 0.57 |  |  |
| Sexual orientation | | | | | | | | |
|  | Homosexual | 179 | 542 | 33.03 | ref | -- |  |  |
|  | Bisexual | 481 | 1422 | 33.82 | 1.02 (0.86, 1.22) | 0.78 |  |  |
| Owns a cell phone | | | | | | | | |
|  | No | 38 | 55 | 68.49 | **2.21 (1.59, 3.07)** | <.01 | **1.62 (1.14, 2.30)** | <.01 |
|  | Yes | 622 | 1906 | 32.64 | ref | -- | -- | -- |
| Number of male sexual partners in past year | | | | | | | | |
|  | 0-4 | 309 | 956 | 32.32 | ref | -- |  |  |
|  | 5-9 | 177 | 486 | 36.41 | 1.15 (0.95, 1.38) | 0.45 |  |  |
|  | 10+ | 164 | 512 | 32.04 | 0.99 (0.82, 1.20) | 0.93 |  |  |
| Receptive and/or insertive anal sexual practices in the past year | | | | | | | | |
|  | RAI only | 109 | 370 | 29.49 | ref |  |  |  |
|  | IAI only | 207 | 562 | 36.83 | 1.24 (0.99, 1.57) | 0.07 |  |  |
|  | IAI and RAI | 333 | 1019 | 32.67 | 1.12 (0.90, 1.39) | 0.30 |  |  |
| Condom used at last anal sex with male partner | | | | | | | | |
|  | No | 231 | 673 | 34.34 | 1.04 (0.88, 1.22) | 0.67 |  |  |
|  | Yes | 429 | 1285 | 33.37 | ref | -- |  |  |
| Sold sex in the past year | | | | | | | | |
|  | No | 358 | 1233 | 29.03 | ref | -- | -- | -- |
|  | Yes | 297 | 724 | 41.01 | **1.42 (1.22, 1.66)** | <.01 | **1.35 (1.15, 1.60)** | <.01 |
| Bought sex in the past year | | | | | | | | |
|  | No | 475 | 1410 | 33.69 | ref |  |  |  |
|  | Yes | 180 | 550 | 32.70 | 0.97 (0.82, 1.16) | 0.76 |  |  |
| Ever tested for HIV | | | | | | | | |
|  | No | 174 | 351 | 49.53 | **1.68 (1.41, 2.00)** | <.01 | **1.43 (1.19, 1.72)** | <.01 |
|  | Yes | 487 | 1611 | 30.24 | ref | -- | -- | -- |
| At least somewhat worried about HIV in past year | | | | | | | | |
|  | No | 447 | 1268 | 35.24 | 1.16 (0.99, 1.37) | 0.07 |  |  |
|  | Yes | 212 | 694 | 30.55 | ref | -- |  |  |
| Ever been verbally harassed for being MSM | | | | | | | | |
|  | No | 459 | 1380 | 33.25 | ref | -- |  |  |
|  | Yes | 203 | 585 | 34.71 | 1.02 (0.86, 1.20) | 0.83 |  |  |
| Ever disclosed MSM status to healthcare worker | | | | | | | | |
|  | No | 421 | 1217 | 34.60 | 1.12 (0.95, 1.31) | 0.17 |  |  |
|  | Yes | 238 | 746 | 31.91 | ref | -- |  |  |
| Ever been afraid to access health services | | | | | | | | |
|  | No | 442 | 1287 | 34.35 | 1.07 (0.91, 1.25) | 0.43 |  |  |
|  | Yes | 221 | 678 | 32.59 | ref | -- |  |  |
| Friends with whom socialize are MSM and heterosexual | | | | | | | | |
|  | No | 49 | 132 | 36.99 | ref | -- |  |  |
|  | Yes | 610 | 1830 | 33.34 | 0.91 (0.68, 1.22) | 0.53 |  |  |
| Trusts the majority of other MSM they know | | | | | | | | |
|  | No | 275 | 980 | 28.07 | ref | -- | -- | -- |
|  | Yes | 382 | 981 | 38.92 | **1.37 (1.18, 1.60)** | <.01 | **1.33 (1.13, 1.55)** | <.01 |
| *Chlamydia trachomatis* | | | | | | | | |
|  | Negative | 548 | 1644 | 33.33 | 1.01 (0.82, 1.25) | 0.90 |  |  |
|  | Positive | 103 | 313 | 32.90 | ref | -- |  |  |
| *Neisseria gonorrhoeae* | | | | | | | | |
|  | Negative | 538 | 1503 | 35.80 | **1.44 (1.18, 1.77)** | <.01 | **1.44 (1.17, 1.78)** | <.01 |
|  | Positive | 113 | 455 | 24.85 | ref | -- | -- | -- |
| Abbreviations: LTFU, loss-to-follow-up; PY, person-years; HR, hazard ratio; CI, confidence interval; RAI, receptive anal intercourse; IAI, insertive anal intercourse; MSM, men who have sex with men. | | | | | | | | |
| Bolding indicates p<0.05 | | | | | | | | |

| *Supplemental Table 3. Visit adherence among participants enrolled in TRUST/RV368 by demographic, behavioral, and clinical characteristics: cisgender men* | | | | | | |
| --- | --- | --- | --- | --- | --- | --- |
| **Characteristic** | | **Visit adherence rate**  **(95% CI)** | **Crude**  **RR (95% CI)** | ***p*** | **Adjusted**  **RR (95% CI)** | ***p*** |
| Overall | | 0.61 (0.59, 0.63) | -- | -- |  |  |
| HIV | | | | | |  |
|  | At risk | 0.51 (0.48, 0.53) | **0.72 (0.68, 0.75)** | <.01 | **0.79 (0.74, 0.84)** | <.01 |
|  | Living with HIV | 0.71 (0.69, 0.74) | ref | -- | -- | -- |
| Age (years) | | | | | |  |
|  | 16-19 | 0.51 (0.47, 0.56) | **0.78 (0.71, 0.86)** | <.01 | 0.94 (0.85, 1.04) | 0.25 |
|  | 20-24 | 0.59 (0.57, 0.62) | **0.90 (0.85, 0.96)** | <.01 | **0.94 (0.88, 1.00)** | 0.04 |
|  | 25+ | 0.66 (0.63, 0.68) | ref | -- | -- | -- |
| Study site | | | | | |  |
|  | Abuja | 0.58 (0.56, 0.60) | ref | -- |  |  |
|  | Lagos | 0.68 (0.65, 0.71) | **1.17 (1.11, 1.25)** | <.01 |  |  |
| Education | | | | | |  |
|  | ≤ high school | 0.58 (0.55, 0.60) | **0.86 (0.81, 0.91)** | <.01 |  |  |
|  | > high school | 0.67 (0.64, 0.70) | ref | -- |  |  |
| Employment status | | | | | |  |
|  | Unemployed | 0.64 (0.60, 0.68) | ref | -- |  |  |
|  | Employed/student | 0.61 (0.59, 0.63) | 0.95 (0.89, 1.02) | 0.17 |  |  |
| Sexual orientation | | | | | |  |
|  | Homosexual | 0.63 (0.60, 0.67) | ref | -- |  |  |
|  | Bisexual | 0.61 (0.58, 0.63) | **0.95 (0.90, 1.02)** | 0.15 |  |  |
| Owns a cell phone | | | | | | |
|  | No | 0.41 (0.35, 0.49) | **0.66 (0.56, 0.78)** | <.01 | 0.85 (0.71, 1.01) | 0.07 |
|  | Yes | 0.62 (0.60, 0.64) | ref | -- | -- | -- |
| Number of male sexual partners in the past year | | | | | |  |
|  | 0-4 | 0.62 (0.60, 0.65) | ref | -- |  |  |
|  | 5-9 | 0.60 (0.57, 0.64) | 0.97 (0.90, 1.04) | 0.37 |  |  |
|  | 10+ | 0.62 (0.59, 0.66) | 1.00 (0.94, 1.08) | 0.91 |  |  |
| Receptive and/or insertive anal sexual practices in the past year | | | | | |  |
|  | RAI only | 0.65 (0.61, 0.70) | ref | -- | -- | -- |
|  | IAI only | 0.57 (0.54, 0.61) | **0.88 (0.81, 0.96)** | <.01 | **0.90 (0.82, 0.99)** | 0.03 |
|  | IAI and RAI | 0.63 (0.60, 0.65) | 0.96 (0.89, 1.04) | 0.31 | 0.94 (0.87, 1.01) | 0.11 |
| Condom used at last anal sex with male partner | | | | | |  |
|  | No | 0.59 (0.56, 0.62) | 0.95 (0.90, 1.01) | 0.13 |  |  |
|  | Yes | 0.62 (0.60, 0.64) | ref | -- |  |  |
| Sold sex in the past year | | | | | |  |
|  | No | 0.66 (0.64, 0.68) | ref | -- | -- | -- |
|  | Yes | 0.55 (0.52, 0.58) | **0.83 (0.78, 0.88)** | <.01 | **0.87 (0.82, 0.93)** | <.01 |
| Bought sex in the past year | | | | | | |
|  | No | 0.62 (0.60, 0.64) | ref | **--** |  |  |
|  | Yes | 0.60 (0.57, 0.64) | 0.98 (0.92, 1.04) | 0.46 |  |  |
| Ever tested for HIV | | | | | | |
|  | No | 0.44 (0.41, 0.48) | **0.68 (0.63, 0.73)** | <.01 | **0.75 (0.69, 0.81)** | <.01 |
|  | Yes | 0.66 (0.64, 0.68) | ref | -- | -- | -- |
| At least somewhat worried about HIV in the past year | | | | | |  |
|  | No | 0.60 (0.58, 0.62) | **0.94 (0.88, 1.00)** | 0.03 |  |  |
|  | Yes | 0.64 (0.61, 0.67) | ref | -- |  |  |
| Ever been verbally harassed for being MSM | | | | | |  |
|  | No | 0.61 (0.59, 0.63) | ref | -- |  |  |
|  | Yes | 0.62 (0.58, 0.65) | 1.01 (0.95, 1.08) | 0.78 |  |  |
| Ever disclosed MSM status to healthcare worker | | | | | |  |
|  | No | 0.59 (0.57, 0.61) | **0.91 (0.85, 0.97)** | <.01 |  |  |
|  | Yes | 0.65 (0.62, 0.68) | ref | -- |  |  |
| Ever been afraid to access health services | | | | | |  |
|  | No | 0.59 (0.57, 0.61) | **0.91 (0.85, 0.96)** | <.01 |  |  |
|  | Yes | 0.66 (0.63, 0.68) | ref | -- |  |  |
| Friends with whom socialize are MSM and heterosexual | | | | | |  |
|  | No | 0.52 (0.46, 0.59) | ref | -- | -- | -- |
|  | Yes | 0.62 (0.60, 0.64) | **1.20 (1.05, 1.36)** | <.01 | 1.13 (0.99, 1.29) | 0.06 |
| Trusts the majority of other MSM they know | | | | | |  |
|  | No | 0.67 (0.64, 0.70) | ref | -- | -- | -- |
|  | Yes | 0.56 (0.54, 0.59) | **0.84 (0.79, 0.89)** | <.01 | **0.88 (0.83, 0.93)** | <.01 |
| *Chlamydia trachomatis* | | | | | |  |
|  | Negative | 0.62 (0.60, 0.64) | 1.00 (0.92, 1.08) | 0.90 |  |  |
|  | Positive | 0.62 (0.58, 0.66) | ref | -- |  |  |
| *Neisseria gonorrhoeae* | | | | | |  |
|  | Negative | 0.60 (0.58, 0.62) | **0.87 (0.82, 0.93)** | <.01 | **0.89 (0.83, 0.96)** | <.01 |
|  | Positive | 0.69 (0.65, 0.73) | ref | -- | -- | -- |
| Abbreviations: RR, risk ratio; CI, confidence interval; RAI, receptive anal intercourse; IAI, insertive anal intercourse; MSM, men who have sex with men*.*  Bolding indicates p<0.05 | | | | | | |

| *Supplemental Table 4. Loss-to-follow-up among participants enrolled in TRUST/RV368 by demographic, behavioral, and clinical characteristics: transgender women* | | | | | | | | |
| --- | --- | --- | --- | --- | --- | --- | --- | --- |
|  |  |  |  |  |  |  |  |  |
| **Characteristic** | | **LTFU n** | **PY** | **LTFU rate** | **Crude**  **HR (95% CI)** | ***p*** | **Adjusted**  **HR (95% CI)** | ***p*** |
| Overall | | 81 | 275 | 29.45 | -- | -- |  |  |
| HIV | | | | | | | | |
|  | At risk | 32 | 90 | 35.40 | 1.39 (0.89, 2.18) | 0.14 | 1.16 (0.71, 1.91) | 0.55 |
|  | Living with HIV | 49 | 1136 | 24.21 | ref | -- | -- | -- |
| Age (years) | |  |  |  |  |  |  |  |
|  | 16-19 | 15 | 48 | 31.31 | 1.18 (0.63, 2.22) | 0.61 | 0.95 (0.47, 1.90) | 0.30 |
|  | 20-24 | 39 | 130 | 29.90 | 1.09 (0.67, 1.79) | 0.72 | 1.00 (0.59, 1.68) | 0.99 |
|  | 25+ | 27 | 97 | 27.92 | ref | -- | -- | -- |
| Study site | | | | | | | | |
|  | Abuja | 46 | 145 | 31.66 | ref | -- |  |  |
|  | Lagos | 35 | 130 | 26.97 | 0.88 (0.56, 1.37) | 0.57 |  |  |
| Education | | | | | | | | |
|  | ≤ high school | 59 | 199 | 29.67 | 1.06 (0.65, 1.73) | 0.82 |  |  |
|  | > high school | 227 | 817 | 27.77 | ref | -- |  |  |
| Employment status | | | | | | | | |
|  | Unemployed | 16 | 82 | 19.54 | ref | -- |  |  |
|  | Employed/ student | 65 | 193 | 33.65 | 1.71 (0.99, 2.97) | 0.06 |  |  |
| Sexual orientation | | | | | | | | |
|  | Homosexual | 41 | 161 | 25.50 | ref | -- |  |  |
|  | Bisexual | 40 | 113 | 35.52 | 1.38 (0.89, 2.14) | 0.15 |  |  |
| Owns a cell phone | | | | | | | | |
|  | No | 8 | 12 | 68.73 | **2.60 (1.24, 5.48)** | <.01 | **3.29 (1.52, 7.09)** | <.01 |
|  | Yes | 73 | 263 | 27.71 | ref | -- | -- | -- |
| Number of male sexual partners in past year | | | | | | | | |
|  | 0-4 | 31 | 104 | 29.84 | ref | -- |  |  |
|  | 5-9 | 22 | 83 | 26.44 | 0.90 (0.52, 1.56) | 0.71 |  |  |
|  | 10+ | 27 | 87 | 30.88 | 1.06 (0.63, 1.79) | 0.82 |  |  |
| Receptive and/or insertive anal sexual practices in the past year | | | | | | | | |
|  | RAI only | 33 | 140 | 23.59 | ref |  |  |  |
|  | IAI only | 3 | 10 | 30.12 | 1.37 (0.42, 4.48) | 0.60 |  |  |
|  | IAI and RAI | 43 | 124 | 34.63 | 1.46 (0.93, 2.31) | 0.10 |  |  |
| Condom used at last anal sex with male partner | | | | | | | | |
|  | No | 32 | 117 | 27.43 | 0.92 (0.59, 1.45) | 0.73 |  |  |
|  | Yes | 47 | 157 | 29.87 | ref | -- |  |  |
| Sold sex in the past year | | | | | | | | |
|  | No | 32 | 117 | 27.43 | ref | -- | -- | -- |
|  | Yes | 47 | 157 | 29.87 | 1.26 (0.79, 1.99) | 0.33 | 1.37 (0.84, 2.21) | 0.20 |
| Bought sex in the past year | | | | | | | | |
|  | No | 60 | 195 | 30.84 | ref |  |  |  |
|  | Yes | 20 | 80 | 25.00 | 0.81 (0.48, 1.34) | 0.41 |  |  |
| Ever tested for HIV | | | | | | | | |
|  | No | 21 | 49 | 42.96 | 1.60 (0.97, 2.63) | 0.07 | **1.92 (1.08, 3.40)** | 0.03 |
|  | Yes | 60 | 226 | 26.53 | ref | -- | -- | -- |
| At least somewhat worried about HIV in past year | | | | | | | | |
|  | No | 42 | 150 | 27.98 | 0.96 (0.62, 1.50) | 0.87 |  |  |
|  | Yes | 38 | 124 | 30.55 | ref | -- |  |  |
| Ever been verbally harassed for being MSM | | | | | | | | |
|  | No | 43 | 140 | 30.26 | ref | -- |  |  |
|  | Yes | 38 | 135 | 28.19 | 0.93 (0.60, 1.45) | 0.76 |  |  |
| Ever disclosed MSM status to healthcare worker | | | | | | | | |
|  | No | 54 | 176 | 30.77 | 1.14 (0.71, 1.83) | 0.58 |  |  |
|  | Yes | 26 | 99 | 26.26 | ref | -- |  |  |
| Ever been afraid to access health services | | | | | | | | |
|  | No | 52 | 153 | 33.98 | 1.45 (0.92, 2.29) | 0.11 |  |  |
|  | Yes | 29 | 122 | 23.77 | ref | -- |  |  |
| Friends with whom socialize are MSM and heterosexual | | | | | | | | |
|  | No | 10 | 19 | 52.03 | ref | -- |  |  |
|  | Yes | 71 | 256 | 27.75 | **0.49 (0.25, 0.96)** | 0.04 |  |  |
| Trusts the majority of other MSM they know | | | | | | | | |
|  | No | 38 | 146 | 26.03 | ref | -- | -- | -- |
|  | Yes | 43 | 129 | 33.31 | 1.31 (0.85, 2.03) | 0.22 | 1.25 (0.77, 2.02) | 0.36 |
| *Chlamydia trachomatis* | | | | | | | | |
|  | Negative | 65 | 220 | 29.53 | 1.06 (0.60, 1.85) | 0.85 |  |  |
|  | Positive | 15 | 54 | 27.61 | ref | -- |  |  |
| *Neisseria gonorrhoeae* | | | | | | | | |
|  | Negative | 57 | 175 | 32.58 | 1.43 (0.88, 2.33) | 0.15 | 1.68 (0.98, 2.87) | 0.06 |
|  | Positive | 23 | 99 | 23.12 | ref | -- | -- | -- |
| Abbreviations: LTFU, loss-to-follow-up; PY, person-years; HR, hazard ratio; CI, confidence interval; RAI, receptive anal intercourse; IAI, insertive anal intercourse; MSM, men who have sex with men. | | | | | | | | |
| Bolding indicates p<0.05 | | | | | | | | |

| *Supplemental Table 5. Visit adherence among participants enrolled in TRUST/RV368 by demographic, behavioral, and clinical characteristics: transgender women* | | | | | | |
| --- | --- | --- | --- | --- | --- | --- |
| **Characteristic** | | **Visit adherence rate**  **(95% CI)** | **Crude**  **RR (95% CI)** | ***p*** | **Adjusted**  **RR (95% CI)** | ***p*** |
| Overall | | 0.66 (0.62, 0.71) | -- | -- |  |  |
| HIV | | | | | |  |
|  | At risk | 0.58 (0.51, 0.66) | **0.81 (0.70, 0.95)** | 0.01 | 0.96 (0.81, 1.15) | 0.69 |
|  | Living with HIV | 0.71 (0.69, 0.74) | ref | -- | -- | -- |
| Age (years) | | | | | |  |
|  | 16-19 | 0.67 (0.56, 0.80) | 0.99 (0.80, 1.23) | 0.93 | 0.99 (0.78, 1.25) | 0.93 |
|  | 20-24 | 0.65 (0.59, 0.73) | 0.97 (0.82, 1.14) | 0.71 | 0.97 (0.82, 1.15) | 0.70 |
|  | 25+ | 0.67 (0.60, 0.76) | ref | -- | -- | -- |
| Study site | | | | | |  |
|  | Abuja | 0.57 (0.51, 0.64) | ref | -- |  |  |
|  | Lagos | 0.77 (0.69, 0.85) | **1.34 (1.15, 1.55)** | <.01 |  |  |
| Education | | | | | |  |
|  | ≤ high school | 0.64 (0.59, 0.70) | 0.89 (0.76, 1.05) | 0.15 |  |  |
|  | > high school | 0.72 (0.63, 0.83) | ref | -- |  |  |
| Employment status | | | | | |  |
|  | Unemployed | 0.77 (0.67, 0.88) | ref | -- |  |  |
|  | Employed/student | 0.63 (0.58, 0.69) | **0.82 (0.70, 0.96)** | 0.01 |  |  |
| Sexual orientation | | | | | |  |
|  | Homosexual | 0.71 (0.64, 0.78) | ref | -- |  |  |
|  | Bisexual | 0.60 (0.53, 0.67) | 0.85 (0.73, 0.99) | 0.03 |  |  |
| Owns a cell phone | | | | | | |
|  | No | 0.44 (0.31, 0.61) | **0.64 (0.45, 0.90)** | 0.01 | **0.64 (0.45, 0.92)** | 0.02 |
|  | Yes | 0.62 (0.60, 0.64) | ref | -- | -- | -- |
| Number of male sexual partners in the past year | | | | | |  |
|  | 0-4 | 0.62 (0.55, 0.70) | ref | -- |  |  |
|  | 5-9 | 0.70 (0.61, 0.80) | 1.13 (0.94, 1.36) | 0.18 |  |  |
|  | 10+ | 0.70 (0.61, 0.79) | 1.12 (0.94, 1.34) | 0.20 |  |  |
| Receptive and/or insertive anal sexual practices in the past year | | | | | |  |
|  | RAI only | 0.73 (0.66, 0.81) | ref | -- | -- | -- |
|  | IAI only | 0.60 (0.41, 0.86) | 0.81 (0.55, 1.19) | 0.29 | 0.80 (0.54, 1.18) | 0.26 |
|  | IAI and RAI | 0.61 (0.55, 0.69) | **0.84 (0.72, 0.97)** | 0.02 | **0.83 (0.71, 0.98)** | 0.03 |
| Condom used at last anal sex with male partner | | | | | |  |
|  | No | 0.66 (0.59, 0.74) | 0.97 (0.84, 1.13) | 0.73 |  |  |
|  | Yes | 0.68 (0.62, 0.74) | ref | -- |  |  |
| Sold sex in the past year | | | | | |  |
|  | No | 0.68 (0.60, 0.77) | ref | -- | -- | -- |
|  | Yes | 0.66 (0.60, 0.72) | 0.97 (0.83, 1.12) | 0.65 | 0.99 (0.85, 1.16) | 0.91 |
| Bought sex in the past year | | | | | | |
|  | No | 0.66 (0.61, 0.72) | ref | **--** |  |  |
|  | Yes | 0.68 (0.59, 0.78) | 1.03 (0.87, 1.21) | 0.76 |  |  |
| Ever tested for HIV | | | | | | |
|  | No | 0.47 (0.39, 0.57) | **0.66 (0.53, 0.81)** | <.01 | **0.66 (0.53, 0.82)** | <.01 |
|  | Yes | 0.71 (0.66, 0.77) | ref | -- | -- | -- |
| At least somewhat worried about HIV in the past year | | | | | |  |
|  | No | 0.60 (0.58, 0.62) | **0.94 (0.88, 1.00)** | 0.03 |  |  |
|  | Yes | 0.64 (0.61, 0.67) | ref | -- |  |  |
| Ever been verbally harassed for being MSM | | | | | |  |
|  | No | 0.63 (0.57, 0.70) | ref | -- |  |  |
|  | Yes | 0.71 (0.64, 0.78) | 1.12 (0.97, 1.30) | 0.12 |  |  |
| Ever disclosed MSM status to healthcare worker | | | | | |  |
|  | No | 0.64 (0.58, 0.70) | 0.88 (0.76, 1.03) | 0.10 |  |  |
|  | Yes | 0.72 (0.64, 0.68) | ref | -- |  |  |
| Ever been afraid to access health services | | | | | |  |
|  | No | 0.61 (0.56, 0.68) | **0.83 (0.72, 0.96)** | 0.01 |  |  |
|  | Yes | 0.74 (0.66, 0.82) | ref | -- |  |  |
| Friends with whom socialize are MSM and heterosexual | | | | | |  |
|  | No | 0.54 (0.41, 0.71) | ref | -- | -- | -- |
|  | Yes | 0.68 (0.63, 0.73) | 1.26 (0.95, 1.68) | 0.11 | 1.26 (0.94, 1.70) | 0.12 |
| Trusts the majority of other MSM they know | | | | | |  |
|  | No | 0.70 (0.63, 0.77) | ref | -- | -- | -- |
|  | Yes | 0.63 (0.56, 0.70) | 0.90 (0.78, 1.04) | 0.16 | 0.96 (0.82, 1.12) | 0.60 |
| *Chlamydia trachomatis* | | | | | |  |
|  | Negative | 0.65 (0.60, 0.71) | 0.91 (0.76, 1.09) | 0.32 |  |  |
|  | Positive | 0.72 (0.61, 0.84) | ref | -- |  |  |
| *Neisseria gonorrhoeae* | | | | | |  |
|  | Negative | 0.62 (0.57, 0.68) | **0.82 (0.70, 0.95)** | <.01 | **0.81 (0.69, 0.95)** | <.01 |
|  | Positive | 0.76 (0.68, 0.86) | ref | -- | -- | -- |
| Abbreviations: RR, risk ratio; CI, confidence interval; RAI, receptive anal intercourse; IAI, insertive anal intercourse; MSM, men who have sex with men*.*  Bolding indicates p<0.05 | | | | | | |

| *Supplemental Table 6. Loss-to-follow-up among participants enrolled in TRUST/RV368 by demographic, behavioral, and clinical characteristics: other or unknown gender* | | | | | | | | |
| --- | --- | --- | --- | --- | --- | --- | --- | --- |
|  |  |  |  |  |  |  |  |  |
| **Characteristic** | | **LTFU n** | **PY** | **LTFU rate** | **Crude**  **HR (95% CI)** | ***p*** | **Adjusted**  **HR (95% CI)** | ***p*** |
| Overall | | 61 | 222 | 27.44 | -- | -- |  |  |
| HIV | | | | | | | | |
|  | At risk | 27 | 52 | 52.08 | **2.54 (1.50, 4.32)** | <.01 | **2.06 (1.17, 3.64)** | 0.01 |
|  | Living with HIV | 34 | 170 | 19.95 | ref | -- | -- | -- |
| Age (years) | |  |  |  |  |  |  |  |
|  | 16-19 | 6 | 19 | 31.61 | 1.57 (0.63, 3.93) | 0.34 | 0.79 (0.27, 2.35) | 0.67 |
|  | 20-24 | 35 | 91 | 38.39 | **2.11 (1.22, 3.65)** | <.01 | 1.51 (0.82, 2.77) | 0.19 |
|  | 25+ | 20 | 112 | 17.84 | ref | -- | -- | -- |
| Study site | | | | | | | | |
|  | Abuja | 43 | 165 | 26.10 | ref | -- |  |  |
|  | Lagos | 18 | 58 | 31.29 | 1.05 (0.60, 1.85) | 0.85 |  |  |
| Education | | | | | | | | |
|  | ≤ high school | 40 | 141 | 28.37 | 1.12 (0.66, 1.90) | 0.67 |  |  |
|  | > high school | 21 | 81 | 25.83 | ref | -- |  |  |
| Employment status | | | | | | | | |
|  | Unemployed | 21 | 51 | 41.24 | ref | -- |  |  |
|  | Employed/ student | 35 | 168 | 20.78 | **0.52 (0.30, 0.90)** | 0.02 |  |  |
| Sexual orientation | | | | | | | | |
|  | Homosexual | 26 | 92 | 28.12 | ref | -- |  |  |
|  | Bisexual | 35 | 130 | 26.96 | 0.93 (0.56, 1.55) | 0.79 |  |  |
| Owns a cell phone | | | | | | | | |
|  | No | 5 | 6 | 85.32 | **2.99 (1.17, 7.59)** | 0.02 | 2.15 (0.80, 5.79) | 0.13 |
|  | Yes | 56 | 211 | 26.51 | ref | -- | -- | -- |
| Number of male sexual partners in past year | | | | | | | | |
|  | 0-4 | 25 | 83 | 30.27 | ref | -- |  |  |
|  | 5-9 | 14 | 68 | 20.49 | 0.70 (0.36, 1.36) | 0.29 |  |  |
|  | 10+ | 21 | 71 | 29.65 | 0.99 (0.55, 1.78) | 0.98 |  |  |
| Receptive and/or insertive anal sexual practices in the past year | | | | | | | | |
|  | RAI only | 13 | 37 | 35.35 | ref |  |  |  |
|  | IAI only | 5 | 17 | 28.92 | 0.86 (0.31, 2.41) | 0.77 |  |  |
|  | IAI and RAI | 40 | 163 | 24.60 | 0.74 (0.39, 1.39) | 0.35 |  |  |
| Condom used at last anal sex with male partner | | | | | | | | |
|  | No | 21 | 64 | 32.74 | 1.27 (0.75, 2.17) | 0.37 |  |  |
|  | Yes | 39 | 156 | 24.96 | ref | -- |  |  |
| Sold sex in the past year | | | | | | | | |
|  | No | 26 | 119 | 21.85 | ref | -- | -- | -- |
|  | Yes | 34 | 103 | 33.08 | 1.51 (0.91, 2.52) | 0.11 | 1.36 (0.78, 2.38) | 0.28 |
| Bought sex in the past year | | | | | | | | |
|  | No | 38 | 166 | 22.86 | ref |  |  |  |
|  | Yes | 22 | 56 | 39.63 | **1.74 (1.02, 2.95)** | 0.04 |  |  |
| Ever tested for HIV | | | | | | | | |
|  | No | 10 | 16 | 63.73 | **2.35 (1.18, 4.66)** | 0.01 | 1.70 (0.79, 3.66) | 0.17 |
|  | Yes | 51 | 207 | 24.69 | ref | -- | -- | -- |
| At least somewhat worried about HIV in past year | | | | | | | | |
|  | No | 34 | 132 | 25.73 | 0.84 (0.51, 1.39) | 0.50 |  |  |
|  | Yes | 27 | 90 | 29.96 | ref | -- |  |  |
| Ever been verbally harassed for being MSM | | | | | | | | |
|  | No | 36 | 127 | 28.37 | ref | -- |  |  |
|  | Yes | 25 | 95 | 26.21 | 0.96 (0.58, 1.60) | 0.88 |  |  |
| Ever disclosed MSM status to healthcare worker | | | | | | | | |
|  | No | 39 | 107 | 36.44 | **1.88 (1.11, 3.17)** | 0.02 |  |  |
|  | Yes | 22 | 115 | 19.09 | ref | -- |  |  |
| Ever been afraid to access health services | | | | | | | | |
|  | No | 32 | 142 | 22.56 | 0.65 (0.39, 1.08) | 0.10 |  |  |
|  | Yes | 29 | 80 | 36.07 | ref | -- |  |  |
| Friends with whom socialize are MSM and heterosexual | | | | | | | | |
|  | No | 4 | 10 | 38.24 | ref | -- |  |  |
|  | Yes | 55 | 211 | 26.10 | 0.74 (0.27, 2.07) | 0.57 |  |  |
| Trusts the majority of other MSM they know | | | | | | | | |
|  | No | 30 | 129 | 23.26 | ref | -- | -- | -- |
|  | Yes | 31 | 93 | 33.24 | 1.44 (0.87, 2.39) | 0.16 | 1.32 (0.77, 2.26) | 0.32 |
| *Chlamydia trachomatis* | | | | | | | | |
|  | Negative | 51 | 176 | 28.93 | 1.31 (0.66, 2.59) | 0.44 |  |  |
|  | Positive | 10 | 46 | 21.98 | ref | -- |  |  |
| *Neisseria gonorrhoeae* | | | | | | | | |
|  | Negative | 49 | 166 | 29.57 | 1.37 (0.73, 2.58) | 0.33 | 1.70 (0.88, 3.28) | 0.12 |
|  | Positive | 12 | 56 | 21.41 | ref | -- | -- | -- |
| Abbreviations: LTFU, loss-to-follow-up; PY, person-years; HR, hazard ratio; CI, confidence interval; RAI, receptive anal intercourse; IAI, insertive anal intercourse; MSM, men who have sex with men. | | | | | | | | |
| Bolding indicates p<0.05 | | | | | | | | |

| *Supplemental Table 7. Visit adherence among participants enrolled in TRUST/RV368 by demographic, behavioral, and clinical characteristics: other or unknown gender* | | | | | | |
| --- | --- | --- | --- | --- | --- | --- |
| **Characteristic** | | **Visit adherence rate**  **(95% CI)** | **Crude**  **RR (95% CI)** | ***p*** | **Adjusted**  **RR (95% CI)** | ***p*** |
| Overall | | 0.66 (0.61, 0.72) | -- | -- |  |  |
| HIV | | | | | |  |
|  | At risk | 0.52 (0.45, 0.61) | **0.70 (0.58, 0.85)** | <.01 | **0.79 (0.64, 0.98)** | 0.03 |
|  | Living with HIV | 0.71 (0.69, 0.74) | ref | -- | -- | -- |
| Age (years) | | | | | |  |
|  | 16-19 | 0.64 (0.50, 0.82) | 0.81 (0.62, 1.07) | 0.13 | 0.94 (0.68, 1.30) | 0.72 |
|  | 20-24 | 0.54 (0.47, 0.63) | **0.69 (0.57, 0.82)** | <.01 | **0.76 (0.62, 0.93)** | <.01 |
|  | 25+ | 0.79 (0.71, 0.89) | ref | -- | -- | -- |
| Study site | | | | | |  |
|  | Abuja | 0.65 (0.58, 0.72) | ref | -- |  |  |
|  | Lagos | 0.71 (0.61, 0.83) | 1.10 (0.91, 1.32) | 0.32 |  |  |
| Education | | | | | |  |
|  | ≤ high school | 0.65 (0.59, 0.73) | 0.95 (0.80, 1.14) | 0.59 |  |  |
|  | > high school | 0.68 (0.60, 0.79) | ref | -- |  |  |
| Employment status | | | | | |  |
|  | Unemployed | 0.57 (0.48, 0.67) | ref | -- |  |  |
|  | Employed/student | 0.73 (0.66, 0.81) | **1.29 (1.07, 1.57)** | <.01 |  |  |
| Sexual orientation | | | | | |  |
|  | Homosexual | 0.66 (0.58, 0.75) | ref | -- |  |  |
|  | Bisexual | 0.67 (0.60, 0.74) | 1.01 (0.85, 1.20) | 0.88 |  |  |
| Owns a cell phone | | | | | | |
|  | No | 0.45 (0.28, 0.71) | 0.67 (0.42, 1.07) | 0.09 | 0.88 (0.53, 1.47) | 0.63 |
|  | Yes | 0.62 (0.60, 0.64) | ref | -- | -- | -- |
| Number of male sexual partners in the past year | | | | | |  |
|  | 0-4 | 0.68 (0.60, 0.78) | ref | -- |  |  |
|  | 5-9 | 0.69 (0.59, 0.81) | 1.02 (0.83, 1.24) | 0.88 |  |  |
|  | 10+ | 0.62 (0.53, 0.73) | 0.91 (0.74, 1.12) | 0.39 |  |  |
| Receptive and/or insertive anal sexual practices in the past year | | | | | |  |
|  | RAI only | 0.63 (0.52, 0.76) | ref | -- | -- | -- |
|  | IAI only | 0.67 (0.50, 0.89) | 1.07 (0.75, 1.51) | 0.72 | 1.13 (0.78, 1.64) | 0.51 |
|  | IAI and RAI | 0.69 (0.62, 0.69) | 1.10 (0.88, 1.36) | 0.41 | 1.04 (0.82, 1.32) | 0.76 |
| Condom used at last anal sex with male partner | | | | | |  |
|  | No | 0.63 (0.54, 0.74) | 0.93 (0.78, 1.12) | 0.47 |  |  |
|  | Yes | 0.68 (0.61, 0.75) | ref | -- |  |  |
| Sold sex in the past year | | | | | |  |
|  | No | 0.69 (0.62, 0.78) | ref | -- | -- | -- |
|  | Yes | 0.64 (0.56, 0.73) | 0.92 (0.78, 1.09) | 0.36 | 1.01 (0.84, 1.22) | 0.91 |
| Bought sex in the past year | | | | | | |
|  | No | 0.71 (0.64, 0.78) | ref | **--** |  |  |
|  | Yes | 0.58 (0.49, 0.68) | **0.81 (0.67, 0.99)** | 0.04 |  |  |
| Ever tested for HIV | | | | | | |
|  | No | 0.47 (0.35, 0.64) | **0.69 (0.50, 0.94)** | 0.02 | 0.81 (0.57, 1.15) | 0.25 |
|  | Yes | 0.71 (0.66, 0.77) | ref | -- | -- | -- |
| At least somewhat worried about HIV in the past year | | | | | |  |
|  | No | 0.68 (0.61, 0.76) | 1.06 (0.89, 1.26) | 0.51 |  |  |
|  | Yes | 0.64 (0.56, 0.73) | ref | -- |  |  |
| Ever been verbally harassed for being MSM | | | | | |  |
|  | No | 0.67 (0.60, 0.75) | ref | -- |  |  |
|  | Yes | 0.66 (0.57, 0.75) | 0.98 (0.83, 1.17) | 0.84 |  |  |
| Ever disclosed MSM status to healthcare worker | | | | | |  |
|  | No | 0.62 (0.55, 0.70) | 0.87 (0.73, 1.02) | 0.09 |  |  |
|  | Yes | 0.72 (0.64, 0.81) | ref | -- |  |  |
| Ever been afraid to access health services | | | | | |  |
|  | No | 0.71 (0.64, 0.79) | **1.22 (1.02, 1.46)** | 0.03 |  |  |
|  | Yes | 0.58 (0.50, 0.68) | ref | -- |  |  |
| Friends with whom socialize are MSM and heterosexual | | | | | |  |
|  | No | 0.59 (0.41, 0.84) | ref | -- | -- | -- |
|  | Yes | 0.68 (0.62, 0.74) | 1.16 (0.80, 1.67) | 0.44 | 1.20 (0.81, 1.77) | 0.36 |
| Trusts the majority of other MSM they know | | | | | |  |
|  | No | 0.70 (0.63, 0.79) | ref | -- | -- | -- |
|  | Yes | 0.62 (0.55, 0.70) | 0.88 (0.74, 1.04) | 0.15 | 0.99 (0.83, 1.18) | 0.60 |
| *Chlamydia trachomatis* | | | | | |  |
|  | Negative | 0.65 (0.59, 0.71) | 0.88 (0.71, 1.08) | 0.21 |  |  |
|  | Positive | 0.74 (0.61, 0.88) | ref | -- |  |  |
| *Neisseria gonorrhoeae* | | | | | |  |
|  | Negative | 0.66 (0.60, 0.73) | 0.99 (0.81, 1.21) | 0.95 | 0.94 (0.75, 1.16) | 0.54 |
|  | Positive | 0.67 (0.56, 0.79) | ref | -- | -- | -- |
| Abbreviations: RR, risk ratio; CI, confidence interval; RAI, receptive anal intercourse; IAI, insertive anal intercourse; MSM, men who have sex with men*.*  Bolding indicates p<0.05 | | | | | | |
